# Doctors’ maintenance of professional competence: a qualitative study informed by the theory of planned behaviour

**DOI:** 10.1186/s12913-021-06438-9

**Published:** 2021-05-03

**Authors:** Anél Wiese, Emer Galvin, Janet O’Farrell, Jantze Cotter, Deirdre Bennett

**Affiliations:** 1 Medical Education Unit, School of Medicine, University College Cork, Cork, Ireland; 2 Medical Education Unit, School of Medicine, University College Cork, Cork, Ireland; 3 Medical Council of Ireland, Dublin, Ireland; 4 Medical Council of Ireland, Dublin, Ireland; 5 Medical Education Unit, School of Medicine, University College Cork, Cork, Ireland

**Keywords:** Health services research, Maintenance of professional competence, Medical regulation, Qualitative research, The theory of planned behaviour

## Abstract

**Background:**

Medical regulators worldwide have implemented programmes of maintenance of professional competence (MPC) to ensure that doctors, throughout their careers, are up to date and fit to practice. The introduction of MPC required doctors to adopt a range of new behaviours. Despite high enrolment rates on these programmes, it remains uncertain whether doctors engage in the process because they perceive benefits like improvements in their practice and professional development or if they solely meet the requirements to retain medical registration. In this study, we aimed to explore the relationship between doctors’ beliefs, intention and behaviour regarding MPC through the lens of the Theory of Planned Behaviour (TPB) to make explicit the factors that drive meaningful engagement with the process.

**Methods:**

We conducted a qualitative study using semi-structured interviews. From a pool of 1258 potential participants, we purposively selected doctors from multiple specialities, age groups, and locations across Ireland. We used thematic analysis, and the TPB informed the analytic coding process.

**Results:**

Forty-one doctors participated in the study. The data analysis revealed doctors’ intention and behaviour and the factors that shape their engagement with MPC. We found that attitudes and beliefs about the benefits and impact of MPC mediated the nature of doctors’ engagement with the process. Some participants perceived positive changes in practice and other gains from participating in MPC, which facilitated committed engagement with the process. Others believed MPC was unfair, unnecessary, and lacking any benefit, which negatively influenced their intention and behaviour, and that was demonstrated by formalistic engagement with the process. Although participants with positive and negative attitudes shared perceptions about barriers to participation, such perceptions did not over-ride strongly positive beliefs about the benefits of MPC. While the requirements of the regulator strongly motivated doctors to participate in MPC, beliefs about patient expectations appear to have had less impact on intention and behaviour.

**Conclusions:**

The findings of this study broaden our understanding of the determinants of doctors’ intention and behaviour regarding MPC, which offers a basis for designing targeted interventions. While the barriers to engagement with MPC resonate with previous research findings, our findings challenge critical assumptions about enhancing doctors’ engagement with the process. Overall, our results suggest that focused policy initiatives aimed at strengthening the factors that underpin the intention and behaviour related to committed engagement with MPC are warranted.

**Supplementary Information:**

The online version contains supplementary material available at 10.1186/s12913-021-06438-9.

## Background

Regulation of the medical profession has become an important contemporary issue amidst growing demand to ensure that doctors, throughout their careers, have the knowledge, skills, and attitudes necessary for safe and effective practice. To this end, medical regulators worldwide have adopted continuous evaluative processes that require doctors to periodically demonstrate that they are up to date and fit to practice [1, 2]. The terminology used to define and doctors’ responsibilities related to these programmes vary across jurisdictions [2]. This paper will refer to this process as the maintenance of professional competence (MPC). Requirements vary but, in general, involve participation in continuing professional development (CPD), patient and peer feedback, and audits or quality improvement projects on an annual or multi-annual basis [3, 4].

While it has been demonstrated that MPC has engendered greater doctor engagement with clinical governance systems [5], assessing and quantifying its impact on professional behaviour and patient outcomes have been more challenging [6]. Instead, there has been a stronger focus on doctors’ and other stakeholders’ experiences to determine their attitudes and identify barriers to meeting the requirements of MPC [6–8]. Evaluations of MPC have, in turn, led to ongoing improvements, fine-tuning and promotion of the process to make it more easily accessible to doctors [9]. There is also some evidence that CPD and patient and peer feedback effectively maintain and improve knowledge, skills, and attitudes [10, 11]. Even so, there has been discontent amongst some doctors about MPC because they have not found it useful to their professional development and the time, effort and expense involved outweighed any perceived benefit [7, 8, 12]. Doctors’ trust in the system may be further undermined by the ongoing debate about the fitness of purpose of MPC programmes to ensure that doctors remain competent and educated [13]. Medical regulators must address these issues because prolonged dissatisfaction with the process may lead to unintended and unfavourable consequences. For example, in the United Kingdom (UK), the introduction of revalidation increased the risk of hospital consultants ceasing clinical activity [14], and negative attitudes about the requirements of the process contributed to General Practitioners’ intentions to leave practice [12].

The introduction of MPC requires doctors to adopt a range of new behaviours. Theories of behaviour [15] help explain and understand how people act. Behavioural theories are also helpful for determining the most effective ways to change behaviour. To date theories of behaviour have been underutilised in research on MPC. A small collection of studies informed by theory has provided valuable insights about doctors’ experiences of MPC [16–18]. For example, a recent analysis informed by Normalization Process Theory revealed that MPC had become gradually accepted and embedded over time despite early scepticism and opposition within the medical profession [18]. High enrolment rates in MPC programmes validate these findings [19, 20]. However, it is still unclear whether doctors perceive benefits such as sustained improvement in practice, professional development, and quality of services or conform to the requirements of MPC solely to satisfy regulators, as has been the case previously [21, 22]. Interventions that can enhance deep engagement with MPC may be warranted; such interventions are more likely to be effective when based on theory. The Theory of Planned Behaviour (TPB) is a well-established behavioural theory that provides a complex and comprehensive understanding of the factors influencing behaviour and behavioural intentions such as attitudes toward the behaviour, subjective norms, and perceived behaviour control [23]. We designed a qualitative study informed by the TPB [23] to understand the relationship between doctors’ beliefs about MPC, their intentions and subsequent behaviour related to the process. Based on the findings of this study, regulators, programme designers, and policymakers could promote the factors that support doctors to participate meaningfully in MPC.

## Methods

### Study aims

The specific aims of this study were to 1) explore doctors’ behaviour and beliefs about MPC and 2) identify factors that facilitate or hinder participation in MPC through the lens of the TPB.

### Study setting

The current Irish healthcare system is a two-tier system with a mixture of public and private funding and provision. The financing of Ireland’s healthcare system comes primarily from Government sources, with significant contribution from out-of-pocket payments and private health insurance [24]. The most recent Medical Workforce Intelligence report indicated 23,558 doctors on the medical register at the end of 2019, and 84.6% of doctors registered reported being clinically active in Ireland and employed in various public-funded or public-private mix of services [25]. Twenty-four percent of doctors reported being in a hospital consultant role, 21.8% were General Practitioners, 19.5% non-consultant hospital doctors not in training, 16.9% non-consultant hospital doctors in training, and the rest in community health, management, and public health roles [25].

In Ireland, doctors have been legally mandated to participate in MPC since 2011. The Medical Council, the regulatory body for the medical profession in Ireland, has established a range of Professional Competence Schemes (PCS) to administer the process through 13 national bodies responsible for postgraduate training. The Register of Medical Practitioners is comprised of four divisions in Table 1. Those registered in the general, supervised, and specialist divisions are required to participate in MPC. Medical students, interns or doctors undertaking postgraduate medical training are not required to participate in MPC. To be compliant, each doctor must obtain a minimum of 50 credits (1 credit = 1 h) through CPD activity and complete one quality improvement (clinical/non-clinical) audit per year. A minimum requirement of 20 credits each is set for external and internal CPD, with the remainder coming from personal learning and research/teaching categories. The Medical Council conducts periodic checks that doctors maintain their professional competence and advise doctors who have not met the minimum requirements to take the necessary steps to become compliant. Potentially non-compliance can lead to removal from the register. At the time of this study, all participants were enrolled and complying with the requirements.

**Table 1:** Divisions of the Register of Medical Practitioners

Division	Registrants
**General division**	Medical practitioners who have not completed specialist training and do not occupy a postgraduate training post. Nineteen per cent of doctors in this division are General Practitioners.
**Specialist division**	Medical practitioners who have completed specialist training recognised by the Medical Council and can practise independently as a specialist. Thirty-nine per cent of doctors in this division are General Practitioners.
**Supervised division**	Medical practitioners who have been offered a post that has been approved by the national health service executive, which has specific supervisory arrangements.
**Trainee specialist division**	Trainee specialist registration is specifically for medical practitioners who practise in individually numbered, identifiable postgraduate training posts.

The current study was one of a larger research project aimed at enhancing doctors’ engagement with MPC. The Health Research Board, Ireland, funded the project. The project steering committee consisted of diverse stakeholders, including members of the Irish Medical Council and postgraduate training bodies and a patient safety advocate.

### Theoretical framework

Theory makes sense of social phenomena and provides a specific focus to different aspects of research data and a framework to conduct an analysis. In this study, we focussed on the factors that influence doctors’ intention and behaviour towards MPC participation by applying the theoretical framework provided by the Theory of Planned Behaviour [23]. The TPB is a theory commonly used to predict and understand behaviour and has been successfully applied in various research domains, including health sciences [26]. The TPB asserts that intention is the strongest determinant of behaviour and indicates the effort an individual is likely to devote to performing a behaviour [23]. Intention, in turn, is influenced by three factors: attitudes, subjective norms, and perceived behavioural control [23, 27]. Attitudes represent an overall evaluation of the behaviour, and subjective norms the assessment of whether an individual feels that significant others think they should engage in the behaviour. Perceived behavioural control represents the factors that hinder or promote behaviour. Behaviour follows from intention, and the participants of this study already have been and will continue to engage in the behaviour (participation in MPC). MPC is a continuing process and requires sustained and focussed engagement with regulatory activities. Therefore, this study recognised the sustained intention towards the behaviour that MPC necessitates, with both intention and behaviour feeding into each other on an ongoing basis.

### Study design and sample

We conducted a qualitative study using semi-structured interviews. The ethical approval for this study was obtained from the University College Cork Social Research Ethics Committee. Voluntary participation was by informed consent with assurances of confidentiality and anonymity. A purposive sample of doctors self-selected to participate. Potential participants were identified through a previous cross-sectional survey study [28] about doctors’ attitudes and experiences of MPC, wherein participants were given a choice to participate in the current study. The survey had 5368 respondents [28], of whom 1258 indicated they would be willing to partake in a confidential interview. We purposively selected doctors from multiple specialities, age groups, and locations across Ireland from this group of potential participants.

### Data collection

Study data were collected qualitatively using a semi-structured interview guide developed based on the TPB constructs (supplementary file 1). EG and AW conducted the interviews mostly in person and two by phone. A small gift voucher was offered in token of thanks for participation. The interviews were recorded and transcribed verbatim by a professional transcription agency. The interview transcripts were anonymised, and all identifying information was removed before they were viewed for analysis by the research team.

### Data analysis

Data analysis started inductively using thematic analysis. Thematic analysis is a method of identifying, analysing and reporting patterns or themes within qualitative data [29]. The analysis involved the following steps: familiarisation with the data, generation of initial codes, searching for themes and patterns, reviewing themes and patterns, and defining and naming themes. Data were coded by using NVivo 12 [30] and involved regular discussions amongst the research team. New inductive codes were labelled as they were identified during the coding process. The TPB [23] informed the analytic coding process, acting as a sensitising concept. All codes that potentially included data relating to the study aims and theoretical constructs were recorded. The codes were reviewed one by one, and the findings were systematically ordered under headings. Although patterns of data consistent with the theoretical constructs of the TPB were sought, the researchers remained open to other patterns of meaning within the data. The ordered data were reviewed and revised in discussion amongst team members and were subsequently organised into themes under headings related to the TPB constructs. The final results and themes reflect the constructs and language of the TPB.

## Results

Forty-one doctors participated in this study. Fifty-one percent of participants were female, and 29% were General Practitioners. Participants from all four provinces of Ireland were interviewed. A total of 1276 min of interview audio data was recorded, and the average interview length was 32 min. Table 2 summarises the socio-demographic characteristics of the doctors interviewed.

**Table 2:** Socio-demographic characteristics of study participants

Variables	Frequency (N)	Frequency (%)
**Gender**		
*Female*	21	51.2%
*Male*	20	48.8%
**Age (in years)**		
*25–34*	2	4.9%
*35–44*	10	24.4%
*45–54*	12	29.3%
*55–64*	15	36.6%
*65+*	2	4.9%
**Specialty**		
*General Practice*	11	26.8%
*Psychiatry*	6	14.6%
*Respiratory Medicine*	4	9.8%
*Emergency Medicine*	4	9.8%
*Anaesthesia*	2	4.9%
*Geriatric Medicine*	2	4.9%
*Obstetrics & Gynaecology*	2	4.9%
*Other*	10	24.4%
**Country of Medical Qualification**		
*Ireland*	34	82.9%
*United Kingdom*	4	9.8%
*Other*	3	7.3%
**Health Services Role**		
*Consultant*	23	56.1%
*General Practitioner*	10	24.4%
*Non-Consultant Hospital Doctor*	6	14.6%
*Retired Consultant*	1	2.4%
*Clinical Researcher*	1	2.4%
**Division on the medical register**		
*Specialist*	28	68.3%
*General*	13	31.7%

The data analysis revealed doctors’ intention and behaviour related to MPC and the factors that shape how doctors participate in MPC. Consistent with the TPB, the themes were abstracted into four distinct constructs.

Behavioural beliefs: doctors’ attitudes to participation in MPC

Normative beliefs: doctors’ beliefs about the expectations of the medical regulator, patients, and peers in relation to their involvement in MPC

Perceived control beliefs: doctors’ perceptions about the barriers and facilitators to participation in MPC

Behavioural intention: doctors’ intentions and behaviours towards MPC

### Behavioural beliefs

The analysis identified a breadth of behavioural beliefs ranging from very positive to very negative dispositions. Across individual participants, there were mixed attitudes, and most fell on one side or the other of neutral regarding their feelings about MPC.

Some participants believed that MPC provides a framework that supports and encourages doctors to participate meaningfully and frequently in continuing professional development.

*It gives structure to people in terms of what is needed to keep yourself safe and competent to work in that profession. It keeps people on the ball, and it does reinforce that it is important.* P18 (Male, General Internal Medicine, Non-Consultant Hospital Doctor).

Furthermore, participants thought that the process increases the transparency and accountability of doctors’ learning and development. Some were concerned that if there were no formal MPC programme, many doctors would not keep up their professional development.

*Without the regulation, it would not happen. I think for a lot of people it would not happen at all. It is forcing people to do things that they wouldn’t do otherwise, and I think that’s a good idea.* P9 (Male, Occupational Medicine, Consultant).

Some doctors believed that MPC is a valuable, shared endeavour. One expressed her view on the social and collaborative nature of participation in MPC:

*I think it’s a very positive thing … It encourages you to become engaged in your organisation in terms of internal meetings, audit, quality improvement and then engaging with your colleagues on a national and international basis.* P23 (Female, Radiology, Consultant).

Participants who were positively disposed towards the process also believed that it could positively impact practice and behaviour changes. One explained the impact of MPC as:

*It helps me in my personal behaviour in practice. If it wasn’t there, I think it would be bad, and I would certainly struggle to fill some of the gaps in personal development.* P5 (Male, Respiratory Medicine, Consultant).

Several participants had negative attitudes toward MPC and believed that the process was unfair, unnecessary, and lacking any benefit. Doctors who found participation in MPC to be a negative experience also often did not perceive any impact on their development and practice. Participants who were negatively disposed towards the process believed that doctors were already keeping up to date before the implementation of MPC and that doctors’ lifelong learning did not require external oversight. One doctor explained her dislike of the process as:

*I completely hate it. I think it’s useless. I feel like it does not have the potential to regulate anything because people who want to educate themselves or maintain themselves the continual medical education will do so without this sort of a threat of having to do this every year.* P31 (Female, Neonatology, Consultant).

### Normative beliefs

Overall, participants expressed that the power of the medical regulator and the potential consequences because of non-compliance have a strong influence on their intention and behaviour to participate in MPC. One doctor explained his reason for participation as:

*Since it started, I have fulfilled my obligations, and I would be afraid not to, to be honest with you.* P6 (Male, General Practitioner).

Conversely, patient and peer expectations did not have much of a bearing on participants’ intention and behaviour regarding MPC. Many participants believed that patients were unaware that MPC existed or that doctors were obliged to participate.

*I don’t think patients have any idea of what we do for MPC, and I don’t think they care very much unless there is a problem.* P8 (Female, General Practitioner).

### Perceived control beliefs

Participants identified several barriers and facilitators to participation in MPC. Facilitators to participation were easy to access MPC activities at the workplace, out of hours, and online. One participant explained that her workplace made it easy to meet the requirements:

*I am a full-time employee with the Health Service Executive, so I can easily get the requirements in-house.* P39 (Female, Psychiatry, Consultant).

Another participant found online learning a valuable resource:

*The knowledge is very accessible online now, for example, through the various medical journals making it easy to keep up to date.* P21 (Female, General Practitioner).

Most participants identified barriers to participation in MPC. Common barriers included time, expense, clinical work obligations, and geographical isolation.

One doctor explained the challenge of trying to balance both work and MPC responsibilities:

*I would often find it challenging to juggle my day-to-day work and finding the time to get CPD points …*. *regarding making sure that colleagues are around so that I can leave the hospital and know that the department is safe while I am away.* P14 (Male, Emergency Medicine, Consultant).

Another participant criticised the expense associated with participation in some MPC activities:

*The expense of it is enormous - to attend an international conference now is almost prohibitive even if you have a grant.* P17 (Male, Respiratory Medicine, Consultant).

Participants who were optimistic about MPC were more likely to persevere despite experiencing barriers such as expense. One doctor explained his behaviour as:

*I do more than I am required, but that is because I believe in it, but on the other hand, it is financially quite difficult for me to do it.* P9 (Male, Occupational Medicine, Consultant).

### Behavioural intention and behaviour in MPC

Two patterns of intention and behaviour were identified – *committed engagement* and *formalistic engagement*. Both were underpinned by the attitudes and beliefs described above. All participants were oriented towards one or the other to a greater or lesser extent. For each pattern, excerpts from an exemplar interview are provided to illustrate the relationship between doctors’ beliefs, intentions, and behaviours related to MPC.

#### Committed engagement

This pattern was characterised by an intention to participate in a motivated and reflective manner. It was important for participants oriented toward this pattern to find high-quality and relevant learning opportunities. These doctors thoughtfully planned and structured the MPC activities they engaged with according to their interests and professional developmental needs.

The following excerpt from a Geriatric Medicine consultant’s interview illustrates this pattern of committed engagement. This participant described barriers like the clinical burden and a lack of resources and facilitators, such as working in a large hospital that offers many learning opportunities that make it either more challenging or more straightforward to meet the requirements of MPC (perceived control beliefs). Even with these barriers, he recognised the benefits of participation and had a positive attitude about MPC. He was motivated to participate in various high-quality MPC activities of use to professional development (intentions and behaviour). This excerpt also demonstrates the potential impact of normative beliefs on behaviour. The participant was very aware of the possible negative repercussions of non-compliance from the regulator (Medical Council); however, he did not believe that MPC was at the forefront of patients’ minds. Therefore, normative beliefs related to patient needs may have had less influence on this participant’s behaviour than the medical regulator’s requirements.

**Table:** 

I:	Are there benefits to participating in MPC?
P19:	I would be positive about the benefits of it. I work in a very busy area, so I would view each year that I need to get to a broad spread of meetings, and I would, in a relatively structured way, plan the meetings I want to get to in any given year. I tend to plan over a multi-year cycle to make sure that I get to enough meetings in each area to stay current. I would deliberately be looking for high-quality meetings. Working in a university hospital, I would certainly have plenty of opportunities to meet the requirements. So, I’m positive about the process overall. If you are not registered in a professional competence scheme, then you can’t renew your registration with the medical council and having it hanging over you or the obligation to get it done is a good way of focusing minds.
I:	Do you think it effectively reassures patients and the public that doctors are fit to practice?
P19:	I am not too sure about the awareness that patients would have of it. I think many patients I deal with will expect what a doctor should be and how they should behave, and even how they should dress. I don’t think patients think that far about whether we are licensed and up to date.
I:	Can you think of any barriers to your participation in MPC?
P19:	The clinical burden is probably the biggest constraint regarding accessing courses. And I think the resources are minimal, and there is a lack of a structured approach towards CPD for consultants.

#### Formalistic engagement

This pattern was characterised by participation in MPC that was minimalistic, cynical, and unenthusiastic and involved form-filling, tick-box behaviours to meet the paperwork and credit requirements of MPC. Participants more oriented to this pattern believed it was easy to game the system, strategically participated in meeting the minimum requirements and selected activities based on credit points and effort required rather than learning or practice needs.

The following excerpt from a Consultant Neurologist’s interview exemplifies this pattern. This participant had a negative attitude towards MPC because he felt that the process mostly involved filling forms and did not lead to practice change. Like the previous participant who committedly engaged in the process, this participant also thought that patients lacked recognition of what MPC entailed yet believed that it was essential to meet the requirements to retain Medical Council registration. This participant described how easy it was to meet the minimum standards, and engagement with the process was left to the very last minute. His intention and behaviour were more focussed on completing paperwork than using the MPC activities as a stimulus for professional development. Finally, this excerpt demonstrates how an absence of perceived changes in his practice underpinned this negative attitude, intention, and behaviour towards MPC.

**Table:** 

I:	Are there any benefits to participating in MPC?
P36:	I’m not sure it captures what it’s supposed to try and capture, and that’s the main issue. Does ticking boxes when you go to a meeting tell if you have learnt anything? I’ve no problem getting points, but certainly, other people have problems getting points, and you would wonder whether chasing points is the right way to go.
I:	Do you think participating in MPC effectively reassures patients and the public that doctors are fit to practice?
P36:	I don’t think the public has any idea what MPC means. I’m not even sure that the public knows that there is such a thing as MPC in any detailed way.
I:	Do you think that MPC encourages doctors to continually learn and keep up to date?
P36:	I’m not sure that it does. Well, not in its present format. It certainly forces doctors to do things so that they have points and can tick the box. All it has done for me is bureaucratised what I did anyway. In that, I must collect forms and fill in forms. It’s a tedious waste of time for people like me. I hear that said an awful lot. We all do the things the night before it’s due. It’s very easy to complete this without doing an awful lot of work. But you must have proof of professional development before you can get Medical Council registration.
I:	Has participation in MPC impacted your practice?
P36:	I just have to collect the letters now and certificates and collect my CPD points. Well, I have to do an audit which is always a pain. I don’t enjoy doing that because I feel like I’m forced to do it. And I’m not sure the audit I do, or that other people are doing is really of much value. They do force you to think about a few things. The question is, how much benefit it is. It hasn’t changed my behaviour one bit.

## Discussion

This study explored doctors’ engagement with maintenance of professional competence through the lens of the Theory of Planned Behaviour. We found that attitudes and beliefs about the benefits and impact of MPC mediated the nature of doctors’ engagement with the process. Some participants believed that MPC had value because they perceived positive changes in their own behaviour and practice, which facilitated committed engagement with the process. Others were more sceptical about the benefits of MPC, which negatively influenced their intention and behaviour, and that was demonstrated by formalistic engagement with the process. Although participants with positive and negative attitudes shared perceptions about barriers to participation, such perceptions did not over-ride strongly positive beliefs about the benefits of MPC. While the requirements of the regulator strongly motivated doctors to participate in MPC, beliefs about patient expectations appear to have had less impact on intention and behaviour.

Our findings on the barriers echo previous research findings, which determined that time, expense, access to CPD courses, amongst others, are obstacles to full participation in MPC [6, 28]. Yet, in our study, these barriers did not prevent doctors from supporting MPC if they perceived the benefits of participation. Initiatives and additional supports aimed at reducing the barriers to participation are helpful, and in TPB terms, would increase the perceived control that doctors feel when participating in MPC. However, our findings have demonstrated that the importance of doctors’ faith in the system to add value to professional development and practice should not be underestimated. In our study, doctors who were negatively and positively disposed to MPC described similar barriers to participation in the process. Beliefs that the process added very little or no value to their development, practice, and quality of care appeared to be the key factor in influencing formalistic engagement.

Previous reports have cautioned that some doctors may focus on compliance and process alone [21, 22, 31], and our findings convey a similar message. In this study, sceptical participants met the minimum requirements and expected little meaningful gain from the process. Some of our participants’ opinion that MPC is a bureaucratic activity that has little educational or practical value has been shared by doctors elsewhere [12]. This study has also shown that how doctors understand and conceptualise the purpose and value of MPC is essential for how they intend to participate in the process. There was criticism from some of our participants about the lack of a clear rationale for MPC. Similar critiques about lack of clarity have been raised previously about whether the purpose of MPC is to identify underperforming doctors, raise standards or attempt to achieve both [16, 32].

Parallel to the shift towards greater public accountability from health service organisations, patient and public involvement (PPI) in health care has become central to service improvement [33]. Patient feedback is also a component of a few MPC systems [1]. An evaluation of revalidation in the UK involving both doctor and patient participants showed a lack of familiarity regarding the purpose of MPC and the role of PPI within it [6]. Doctors’ attitudes have also been identified as a significant barrier to PPI in medical performance processes [33]. While most of our participants believed that patients lacked a detailed awareness of MPC, it remains unclear what patients’ expectations are regarding MPC. Further research that explores patients’ and doctors’ understanding of each other’s needs concerning MPC may be worthwhile. Such research may be useful for identifying opportunities for patients, professionals, and policymakers to work collaboratively towards improving regulatory processes and to integrate patients as partners in the design and implementation of MPC.

Successful implementation of complex interventions, like medical regulation, is dependent on a complex network of factors, including the degree to which individuals support, commit and adapt to the process [34, 35]. Individual regulatory systems have tended to implement a top-down, standardised and one-size-fits-all approach [36]. Through legislated authority and sustained pressure, policymakers and regulators have driven doctors to change their behaviour and adopt nation-wide models of MPC. More recently, it has been suggested that a flexible approach tailored to individual, organisational, and professional contexts would help get all doctors to benefit from the process [37]. Additionally, factors that may support doctors’ belief in the value of the process and decrease box-ticking behaviours include embeddedness of MPC activities in practice and better alignment of the requirements with individual goals, motivation, and practice [22]. Implementation and complexity science offers an alternative bottom-up approach [35], which could be a valuable addition to the hierarchical way MPC is currently implemented. A bottom-up approach would involve local arrangements that provide flexibility to a nationally agreed model and engagement from local stakeholders to facilitate ways to improve doctors’ beliefs and participation. A collaborative grassroots-led approach, within a broad regulatory framework, could result in growing acceptance, increased positive attitudes and eventual behaviour and practice change amongst clinicians [35]. Intervention strategies created for changing beliefs and behaviours [38] would suit a bottom-up approach to shifting doctors’ attitudes and behaviour positively towards MPC. Behaviour change interventions often involve targeting individual’s salient beliefs about behaviour, like the beliefs about MPC that we identified through our analysis based on the TPB. Interventions designed to target all three belief categories (attitudes, subjective norms and behavioural control) would be most effective [27].

### Methodological strengths and limitations

The study had a large and diverse sample of doctors; however, the participants were from a single regulatory system. While the literature shows that the issues relating to MPC are similar across jurisdictions, the findings may not be transferable to all settings. Other strengths are that the study design and analysis were informed by theory, the diversity of stakeholders and the inclusivity of both knowledge-users and patient representatives in the project team.

## Conclusions

The study’s findings broaden our understanding of the determinants of doctors’ intention and behaviour regarding participation in MPC, which offers a basis for designing targeted interventions. While the barriers to engagement with MPC resonate with previous research findings, our findings challenge critical assumptions about enhancing doctors’ engagement with the process. Overall, our findings suggest that focused policy initiatives aimed at strengthening the factors that underpin the intention and behaviour related to committed engagement with MPC are warranted.

## Supplementary Information

**Additional file 1: Supplementary file 1.** Interview guide.

## Data Availability

The dataset generated and/or analysed during the current study are not publicly available due to participants not having consented to public availability but are available from the corresponding author on reasonable request.

## Abbreviations

CPD: Continuing Professional Development
MPC: Maintenance of Professional Competence
PCS: Professional Competence Scheme
PPI: Patient and Public Involvement
TPB: Theory of Planned Behaviour
UK: United Kingdom
