# Supplementary material for: Doctors’ maintenance of professional competence: a qualitative study informed by the theory of planned behaviour
Source: BMC Health Serv Res. 2021 May 3;21:419. doi: 10.1186/s12913-021-06438-9 (PMC8094537; doi:10.1186/s12913-021-06438-9)
Supplement: Supplementary file 1 — Additional file 1: Supplementary file 1. Interview guide. [file 12913_2021_6438_MOESM1_ESM.docx]

**Doctors’ maintenance of professional competence. A qualitative study informed by the Theory of Planned Behaviour**

Anel Wiese, Medical Education Unit, School of Medicine, University College Cork, Cork, Ireland. Corresponding author (a.wiese@ucc.ie)

Emer Galvin, Medical Education Unit, School of Medicine, University College Cork, Cork, Ireland

Janet O’Farrell, Medical Council of Ireland, Dublin, Ireland

Jantze Cotter, Medical Council of Ireland, Dublin, Ireland

Deirdre Bennett, Medical Education Unit, School of Medicine, University College Cork, Cork, Ireland

**Supplementary file 1: Interview guide**

| 1. | Could you tell me about your professional background and current role? |
| --- | --- |
| 2. | Can you tell me about your experiences of participating in MPC? |
| 3. | What do you think is the purpose of MPC? |
| 4. | Do you think there are benefits to participating in MPC? |
| 5. | What are your views about the effectiveness of MPC?   - Do you think that it effectively reassures patients and the public that doctors are fit to practice? - Encourages doctors to continually learn and keep up to date? - Raise the standard of practice of doctors? |
| 6. | How has participation in MPC impacted on your practice?   - Has it encouraged you to reflect more on your professional development or to participate in more educational activities? |
| 7. | What are some of the barriers to participation in MPC? |
| 8. | What would support your participation in MPC? |
| 9. | In what activities do you participate to meet the requirements MPC? Tell me about your experience of accessing educational activities?   - Accessibility, relevance to your practice or learning needs, location, etc. |
| 10. | How do you decide what activities to participate in? |
| 13. | Could you tell me about your experience of doing audits or quality improvement? |
| 14. | Do you think doctors receive enough guidance/support about MPC?   - Is there any information or support that you would help you to participate in MPC? |
| 15. | If you could change anything about MPC, what would that be? |
